# Occupational risks for infection with influenza A and B: a national case–control study covering 1 July 2006–31 December 2019

**DOI:** 10.1136/oemed-2022-108755

**Published:** 2023-05-16

**Authors:** Kjell Torén, Maria Albin, Tomas Bergström, Magnus Alderling, Linus Schioler, Maria Åberg

**Affiliations:** 1 Public Health and Community Medicine, University of Gothenburg, Goteborg, Sweden; 2 Occupational and Environmental Medicine Department of Medicine, Sahlgrenska University Hospital, Goteborg, Sweden; 3 Institute of Environmental Medicine, Karolinska Institutet, Stockholm, Sweden; 4 Department of Infectious Diseases/Virology, University of Gothenburg, Goteborg, Sweden; 5 Unit of Occupational Medicine, Institute of Environmental Medicine, Karolinska Institutet, Stockholm, Sweden

**Keywords:** Respiratory System, Epidemiology, Microbiology, Occupational Health, Health Personnel

## Abstract

**Objectives:**

We investigated whether crowded workplaces, sharing surfaces and exposure to infections were factors associated with a positive test for influenza virus.

**Methods:**

We studied 11 300 cases with a positive test for influenza A and 3671 cases of influenza B from Swedish registry of communicable diseases. Six controls for each case were selected from the population registry, with each control being assigned the index date of their corresponding case. We linked job histories to job-exposure matrices (JEMs), to assess different transmission dimensions of influenza and risks for different occupations compared with occupations that the JEM classifies as low exposed. We used adjusted conditional logistic analyses to estimate the ORs for influenza with 95% CI.

**Results:**

The highest odds were for influenza were: regular contact with infected patients (OR 1.64, 95% CI 1.54 to 1.73); never maintained social distance (OR 1.51, 95% CI 1.43 to 1.59); frequently sharing materials/surfaces with the general public (OR 1.41, 95% CI 1.34 to 1.48); close physical proximity (OR 1.54, 95% CI 1.45 to 1.62) and high exposure to diseases or infections (OR 1.54, 95% CI 1.44 to 1.64). There were small differences between influenza A and influenza B. The five occupations with the highest odds as compared with low exposed occupations were: primary care physicians, protective service workers, elementary workers, medical and laboratory technicians, and taxi drivers.

**Conclusions:**

Contact with infected patients, low social distance and sharing surfaces are dimensions that increase risk for influenza A and B. Further safety measures are needed to diminish viral transmission in these contexts.

**WHAT IS ALREADY KNOWN ON THIS TOPIC**

- The frequencies of influenza A and B infections vary across occupational groups. However, there is limited evidence regarding which workplace exposure factors are of importance in relation to an increased risk of infection.

**WHAT THIS STUDY ADDS**

- Contact with infected patients or the general public and short social distance were associated with increased likelihood of having positive test for influenza virus. Primary care physician was found to be the occupation with the highest odds of a positive test for influenza virus.

**HOW THIS STUDY MIGHT AFFECT RESEARCH, PRACTICE OR POLICY**

- There is a need for further safety measures, such as extended vaccination programmes, to diminish viral transmission in workplaces that involve close physical contacts.

## Introduction

Influenza is major global health concern, and higher prevalence of influenza has been reported for settings such as healthcare clinics, prisons, day-care centres and schools.^1^ Influenza can occur in pandemic waves, as was the case with the 2009 influenza pandemic caused by the A(H1N1)pdm09 subtype, although there are also frequently occurring seasonal outbreaks. Influenza A is the dominant cause of seasonal epidemics, whereas the pathogenic character of influenza B is more endemic.^2 3^

Influenza is transmitted via air particles from the mucous membranes of an infected person, by personal contact with an infectious person, or by contact with virus-contaminated surface.^4^ Spreading of the virus via air over longer distances is less common. Workplace exposures may be of importance for the transmission risk of influenza, a phenomenon that has been shown for healthcare workers (HCWs) and other occupations with frequent close contacts with other persons.^5 6^ In a systematic review, HCWs seem to have an at least a doubled risk for influenza A (H1N1) based on studies from the 2009 pandemic.^6^ There are, however, published studies that do not show an increased risk for influenza A virus infection for HCWs.^7–9^ Regarding influenza B viral infections, there are reports of smaller outbreaks in hospital settings.^2 3 10^ In a Spanish case–control study, there was an increased risk for influenza A (H1N1) infection, defined as a positive reverse transcriptase PCR test in certain HCWs with presumed exposure to aerosol-generating procedures.^1^ There was an increased risk for severe influenza (hospitalised cases) among manual workers, as compared with primary care cases (as controls).^11^ In a recent Danish study, the authors a priori selected 10 occupations that are considered to be at risk for hospitalisation due to influenza and compared then with ‘public administration’ as the reference group.^5^ There was no separation between influenza A and B infections. In the same study, an increase in the rate of hospitalisation due to influenza was found among workers in public transportation, followed by garbage and recycling workers and HCWs. These studies indicate that workers who are in close contact with either the general public or with infected patients can be at increased risk for influenza. In this context, studies that differentiate between infections with influenza A and B viruses and subtypes thereof are lacking.

The ongoing COVID-19 pandemic has focused attention on the possibility that the workplace is a key setting for the spread of the SARS-CoV-2 virus.^12–15^ Consequently, several job-exposure matrices (JEMs) have been developed to assess the risk of exposure to SARS-CoV-2.^16 17^ These JEMs are based on categorisation of different occupations according to workplace factors of interest, in this case factors assumed to be linked with risk of SARS-CoV-2 infection. We assume that, in slightly modified forms, these matrices can also be applied to studying the risks for other viral infections, such as those with influenza A and B.^18^

Thus, we hypothesised that in a working population, occupations involving close contacts with coworkers or the general public would be associated with increased odds for influenza. To study this, we apply JEMs matrices that have been developed to assess the risk of exposure to SARS-CoV-2.^16 17^

## Materials and methods

### Establishment of study population

The study population included all cases of influenza A or influenza B virus infection obtained through the system of mandatory reporting of communicable diseases in Sweden, the SmiNet registry, as previously reported by us.^19^ The cases eligible for inclusion were those in the age range of 19–64 years who had a reported positive test for influenza virus, that is, detection of viral ribonucleic acid (RNA) by PCR, (N=14 971), including influenza A (N=11 300) and influenza B (N=3671). We extracted from the SmiNet registry the Swedish personal identity number for each case and the date (index date) when the positive sample was obtained. We selected six living controls for each case, matched for gender, age (case year of birth) and region of residency at the index date from the Swedish Historical National Population Registry (N=66 216). We limited the study to reports received between 1 July 2006 and 31 December 2019. Here, ‘influenza’ is defined as a positive test for influenza virus.

We extracted information from the Swedish national socioeconomic database, called LISA (Longitudinal integration database for health insurance and labour market studies), regarding the highest educational level attained, categorised as: pre high school (up to 9 years), completed high school, or university examination, and country of birth. From LISA, we also obtained information about the annual occupational history for the period of 2005–2019.

### Comorbidities

We used the Swedish National Hospital Discharge Registry and the Swedish Prescribed Drug Registry to identify the following comorbidities based on 10th revision of the International Classification of Diseases (ICD-10) codes: chronic obstructive pulmonary disease (COPD, ICD10 J43–J44); ischaemic heart disease (IHD, ICD10 I20–I25); and diabetes mellitus (ICD E10–E14) during the 5 years preceding the index date. We defined the use of oral and systemic corticosteroids according to the Anatomical Therapeutic Chemical (ATC) codes (ATC H02) if these drugs were dispensed at any time within the 5 years preceding the index date. We also used the Swedish National Hospital Discharge Registry to inform on hospitalisation for any pneumonia, including any hospital stay that included the index date ±7 days. Among the cases, we identified a subset of ‘pneumonia with influenza’, as influenza/viral pneumonia (ICD 10 J09–J12).

### Classification of occupational exposures

The occupation of the individual in the year preceding the index date was classified at the four-digit level according to the ISCO-88 and ISCO-08 codes.^20 21^ We applied two previously described JEMs to assess the risk of becoming infected with the SARS-CoV-2 virus: a European JEM based on expert assessment performed during the COVID-19 pandemic^16^ and a Swedish JEM based on prepandemic US survey data obtained from the UK Office of National Statistics.^17^ The European JEM was designed to capture eight dimensions that were judged to be important for the risk of being infected, divided into: low risk, elevated risk and high risk. All dimensions were compared with no risk—defined as home workers or not working with others. We used the Danish application of the European JEM, which we assumed was the application that would most likely reflect Swedish conditions. Thus, in this study, we applied five of the following categories of risk dimensions:

1. Number of workers in close vicinity to each other. High risk: >30 per day, elevated risk: 10–30 per day and low risk: <10/day.
2. Nature of contacts. High risk: working in workspaces with regular contacts with suspected or diagnosed COVID-19 (for this application, infected patients), elevated risk, working with the general public and low risk: working in workspaces with coworkers only.
3. Contaminated workspaces. High risk: frequently (≥10 times/day) sharing materials/surfaces with the general public, medium risk: sometimes (<10 times/day) sharing material or surfaces with the general public and low risk: frequently (≥10 times/day) sharing materials or surfaces with coworkers, only.
4. Location. High risk: working mostly inside (>4 hours/day), medium risk: working partly inside (1–4 hours/day) and low risk: working mostly outside.
5. Social distancing, that is, the possibility to maintain ≥1 m of social distance. High risk: can never be maintained, elevated risk: cannot always be maintained and low risk: can always be maintained.

We also applied the Swedish JEM that mapped physical proximity and exposure to diseases or infections as previously described.^18^ This JEM has standardised scores for each occupational group, yielding scores in the range of 0–100.

Thus, the scale for physical proximity was as follows:

0—I do not work near other people (>30 m distance).

25—I work with others but not close proximity (eg, private office).

50—I work in slightly close proximity (eg, shared office) to other persons.

75—I work in moderately close proximity (at arm’s length) to other persons.

100—I work in very close proximity (near touching) to other persons.

The scale for daily exposure to diseases or infections at current workplace was as follows:

0—never.

25—at least once a year, but not every month.

50—at least once a month, but not every week.

75—at least once a week, but not daily.

100—daily.

We present this as quartiles of the mean scores for each dimension.

### Statistical methods

We used conditional logistic multivariable regression to calculate the odds for all influenza, influenza A and influenza B, associated with the JEM-defined categories of exposures tested as indicator variables. The basic model (model 1) was only adjusted for matching strata (ie, equivalent to adjusting for gender, age and geographical region and index date). The adjusted model (model 2) included, in addition, education, country of birth, COPD, IHD, diabetes and dispensed corticosteroids. All the JEM-defined categories of exposures were tested in separate models for each exposure. We also analysed the interactions with regard to gender.

We used conditional logistic multivariable regression to calculate the odds for all influenza with pneumonia associated with the JEM-defined categories of exposures tested as indicator variables.

Furthermore, we analysed the risk for all influenza in all occupations (4-digit level) using more than 50 persons with a positive influenza virus test. The reference group in this analysis was defined as occupations classified as having the lowest level of potential exposure to influenza using the European and Swedish JEMs.^15^ All occupations were tested in separate unconditional models for each exposure with adjustments for gender, age and geographical region.

All statistical analyses were performed using the SAS V.9.4 M7 software (SAS), and 95% CIs were calculated.

## Results

The study comprised 14 971 cases of influenza, whereof 11 300 were influenza A and 3671 were influenza B. Diabetes mellitus was a common comorbidity, seen in 5.5% of the cases and 1.6% of the controls. Use of corticosteroids was identified for 16.3% of the cases and for 3.9% of the controls. The prevalences of the occupational exposures are listed in table 1. The most common dimension was working mostly inside, which accounted for 73.7% of the cases and 69.7% of the controls. Additional descriptive data are presented in table 1.

**Table 1:** Characteristics of the cases with influenza and matched controls from the general population of Sweden in the age range 20–65 years in a national case–control study covering 1 July 2006 to 31 December 2019

	Influenza (N=14 971)	Controls (N=66 216)
Men	44.1% (N=6595)	43.8% (N=29 001)
Born in Sweden	73.7% (N=11 035)	83.2% (N=55 114)
Post high school examination	39.7% (N=5947)	43.9% (N=5947)
Chronic obstructive pulmonary disease*	1.7% (N=250)	0.1% (N=82)
Diabetes mellitus*	5.5% (N=828)	1.6% (N=1040)
Ischaemic heart disease*	2.2% (N=794)	0.7% (N=458)
Dispensed corticosteroids	16.3% (N=2442)	3.9% (N=2594)
Occupational exposures or dimensions of transmission and mitigation factors		
No; workers (>30) in close proximity to each other	30.6% (N=4582)	26.8% (N=17 722)
Nature of contacts; regular contacts with infected patients	21.5% (N=3220)	16.3% (N=10 804)
Contaminated workspaces; frequently sharing material/surfaces with general public	46.2% (N=6924)	39.9% (N=26 401)
Location; working mostly inside	73.7% (N=11 041)	69.7% (N=46 174)
Social distancing; Can never be maintained	30.2% (N=4524)	24.3% (N=16 103)
Physical proximity; work very close (near touching) to other persons†	30.6% (N=4573)	24.2% (N=16 015)
Daily exposure to diseases or infections	9.9% (N=1481)	7.6% (n=1 481)

*Hospital-based diagnoses.

†Military personnel not included.

In the basic model, model 1, the odds for influenza A were slightly higher than those for influenza B (table 2). When applying the European JEM, the highest odds were for influenza A and the dimensions of: regular contact with infected patients (OR 1.67, 95% CI 1.55 to 1.77); never maintained social distance (OR 1.54, 95% CI 1.45 to 1.63) and frequently sharing materials/surfaces with the general public (OR 1.43, 95% CI 1.35 to 1.52). This was in accordance with the Swedish JEM, which showed high odds in relation to the fourth quartile of Physical proximity (OR 1.58, 95% CI 1.48 to 1.68) and the fourth quartile of exposure to diseases or infections (OR 1.57, 95% CI 1.46 to 1.69) (table 3).

**Table 2:** Conditional logistic multivariable regression models of influenza (model 1) in relation to the different dimensions of transmission and mitigation factors in a national case–control study covering 1 July 2006 to 31 December 2019

Dimensions of transmission and mitigation factors	Influenza		
	ORs with 95% CI*		
	All (N=14 971)	Influenza A (N=11 300)	Influenza B (N=3671)
No of workers in close proximity to each other†			
<10 per day	1.12 (1.06 to 1.18)	1.14 (1.08 to 1.22)	1.05 (0.95 to 1.17)
10–30 per day	1.32 (1.25 to 1.40)	1.32 (1.24 to 1.41)	1.31 (1.18 to 1.47)
>30 per day	1.40 (1.33 to 1.47)	1.42 (1.33 to 1.51)	1.34 (1.20 to 1.48)
Nature of contacts†			
In workspaces with coworkers only	1.12 (1.06 to 1.18)	1.13 (1.06 to 1.20)	1.08 (0.97 to 1.20)
In workspaces with general public	1.23 (1.17 to 1.30)	1.25 (1.17 to 1.32)	1.18 (1.06 to 1.31)
Regular contacts with infected patients	1.64 (1.54 to 1.73)	1.67 (1.55 to 1.77)	1.57 (1.40 to 1.77)
Contaminated workspaces†			
Frequently sharing material/surfaces with coworkers (≥10 times/day)	1.10 (1.00 to 1.20)	1.14 (1.07 to 1.21)	1.08 (0.97 to 1.20)
Sometimes sharing material/surfaces with general public (<10 times/day)	1.13 (1.02 to 1.24)	1.08 (0.97 to 1.21)	1.05 (0.86 to 1.28)
Frequently sharing materials/surfaces with general public (≥10 times/day)	1.41 (1.34 to 1.48)	1.43 (1.35 to 1.52)	1.34 (1.22 to 1.48)
Location†			
Mostly working outside	1.48 (1.25 to 1.76)	1.51 (1.24 to 1.84)	1.41 (0.99 to 2.02)
Working partly inside	1.16 (1.07 to 1.27)	1.17 (1.06 to 1.29)	1.14 (0.96 to 1.36)
Working mostly inside	1.27 (1.21 to 1.33)	1.29 (1.22 to 1.36)	1.22 (1.11 to 1.33)
Social distancing†			
Always maintained	1.11 (1.06 to 1.17)	1.13 (1.07 to 1.21)	1.06 (0.95 to 1.18)
Not always	1.22 (1.15 to 1.29)	1.22 (1.14 to 1.30)	1.21 (1.09 to 1.36)
Never maintained	1.51 (1.43 to 1.59)	1.54 (1.45 to 1.63)	1.43 (1.28 to 1.59)

*Model adjusted for gender, age and geographic region.

†Compared with homeworkers or working alone.

**Table 3:** Logistic multivariable regression models of influenza (model 2) in relation to the different dimensions of physical proximity and exposure to disease or infections in a national case–control study covering 1 July 2006 to 31 December 2019

Dimensions of physical proximity and exposure to diseases or infections	Influenza		
	ORs with 95% CI*		
	All	Influenza A	Influenza B
Physical proximity			
3rd vs 2nd and 1st	1.12 (1.07 to 1.18)	1.11 (1.09 to 1.22)	1.04 (0.95 to 1.15)
4th vs 2nd and 1st	1.54 (1.45 to 1.62)	1.58 (1.48 to 1.68)	1.42 (1.27 to 1.59)
Exposure to diseases or infections			
2nd vs 1st	1.29 (1.23 to 1.35)	1.27 (1.20 to 1.35)	1.34 (1.21 to 1.47)
3rd vs 1st	1.27 (1.20 to 1.34)	1.28 (1.20 to 1.36)	1.24 (1.11 to 1.38)
4th vs 1st	1.54 (1.44 to 1.64)	1.57 (1.46 to 1.69)	1.43 (1.24 to 1.64)

*Conditional model adjusted for gender, age and geographical region.

Table 4 shows the odds from model 2 with additional adjustments compared with model 1. The odds for influenza A are somewhat lower, although the dimensions of regular contact with infected patients (OR 1.41, 95% CI 1.31 to 1.51) and the fourth quartile of exposure to diseases or infections (OR 1.51, 95% CI 1.40 to 1.63) are still the highest estimates. The odds for influenza B are similar to influenza A, but somewhat lower. Online supplemental table S1 shows the different odds for all influenza for men and women. The estimates were quite similar, but mostly somewhat lower for women than for men.

10.1136/oemed-2022-108755.supp1 Supplementary data

**Table 4:** Conditional logistic multivariable regression models of influenza A or influenza B matched for gender, age and geographical region, and adjusted for education, country of birth, COPD, IHD, diabetes and use of corticosteroids in relation to the different dimensions of transmission and mitigation factors (model 2) in a national case–control study covering 1 July 2006 to 31 December 2019

Dimensions of transmission and mitigation factors	Influenza A and influenza B	
	ORs with 95% CI	
	Influenza A (N=11 300)	Influenza B (N=3671)
No of workers in close proximity to each other*		
<10 per day	1.05 (0.99 to 1.12)	0.98 (0.88 to 1.10)
10–30 per day	1.14 (1.07 to 1.22)	1.17 (1.04 to 1.32)
>30 per day	1.28 (1.20 to 1.37)	1.22 (1.09 to 1.36)
Nature of contacts*		
In workspaces with coworkers only	1.05 (0.99 to 1.12)	1.01 (0.90 to 1.12)
In workspaces with general public	1.12 (1.05 to 1.20)	1.09 (0.97 to 1.21)
Regular contacts with infected patients	1.41 (1.31 to 1.51)	1.37 (1.21 to 1.56)
Contaminated workspaces*		
Frequently sharing material/surfaces with coworkers (≥10 times/day)	1.08 (1.01 to 1.15)	1.03 (0.92 to 1.15)
Sometimes sharing material/surfaces with general public (<10 times/day)	1.06 (0.94 to 1.19)	1.06 (0.86 to 1.30)
Frequently sharing materials/surfaces with general public (≥10 times/day)	1.23 (1.16 to 1.31)	1.19 (1.07 to 1.32)
Location*		
Mostly working outside	1.26 (1.02 to 1.55)	1.16 (0.79 to 1.69)
Working partly inside	1.09 (0.98 to 1.21)	1.10 (0.91 to 1.32)
Working mostly inside	1.16 (1.10 to 1.22)	1.11 (1.01 to 1.22)
Social distancing*		
Always maintained	1.05 (0.97 to 1.14)	1.05 (0.98 to 1.14)
Not always maintained	1.07 (0.98 to 1.16)	1.15 (1.06 to 1.25)
Never maintained	1.40 (1.27 to 1.55)	1.26 (1.18 to 1.36)
Physical proximity		
3rd vs 2nd and 1st	1.11 (1.05 to 1.18)	0.99 (0.89 to 1.09)
4th vs 2nd and 1st	1.39 (1.30 to 1.49)	1.26 (1.12 to 1.41)
Exposure to diseases or infections		
2nd vs 1st	1.13 (1.06 to 1.20)	1.19 (1.08 to 1.32)
3rd vs 1st	1.17 (1.09 to 1.25)	1.17 (1.04 to 1.31)
4th vs 1st	1.51 (1.40 to 1.63)	1.42 (1.23 to 1.65)

*Compared with homeworkers or working alone.

COPD, chronic obstructive pulmonary disease; IHD, ischaemic heart disease.

The odds for influenza with pneumonia were similar to the results for all influenza, with one exception (online supplemental table S2). The odds for the fourth quartile of exposure to diseases or infections (OR 1.05, 95% CI 0.94 to 1.18) was not increased, whereas that for regular contact with infected patients (OR 1.28, 95% CI 1.17 to 1.41) was increased.

Table 5 lists the odds for influenza all occupations with more than 50 cases, as compared with the unexposed control occupations. The five occupations with the highest odds for influenza using model 1 were: primary care physicians (OR 3.21, 95% CI 2.78 to 3.73); protective service workers (OR 2.46, 95% CI 1.78 to 3.41); elementary workers not elsewhere classified (OR 2.43, 95% CI 1.98 to 2.97); medical and laboratory technicians (OR 2.31, 95% CI 1.69 to 3.15) and taxi drivers (OR 1.95, 95% CI 1.54 to 2.45). The five following occupations with high odds were: bus and tram drivers, home-based personal care workers, personal care workers, teachers’ aides and healthcare assistants.

**Table 5:** ORs for influenza in occupations with >50 exposed cases

Occupation	ISCO 2008 No	Exposed cases (N)	OR	95% CI
Primary care physicians	2211	316	3.21	2.78 to 3.73
Protective service workers NEC	5419	53	2.46	1.78 to 3.41
Elementary workers NEC	9629	145	2.43	1.98 to 2.97
Medical and pathology laboratory technicians	3212	57	2.31	1.69 to 3.15
Taxi drivers	8322	104	1.95	1.54 to 2.45
Home-based personal care workers	5322	811	1.91	1.72 to 2.11
Teacher’s aides	5312	143	1.87	1.53 to 2.28
Personal care workers in health services NEC	5329	64	1.87	1.40 to 2.49
Bus and tram drivers	8331	126	1.83	1.49 to 2.26
Nursing professionals	2121	470	1.75	1.55 to 1.97
Healthcare assistants	5321	1289	1.72	1.57 to 1.87
Earth-moving and related plant operators	8342	72	1.71	1.30 to 2.24
Kitchen helpers	9412	297	1.71	1.48 to 1.98
Hairdressers	5141	51	1.69	1.23 to 2.33
Child-care workers	5311	433	1.69	1.49 to 1.91
Government and social benefit officials	3353	66	1.68	1.27 to 2.22
Domestic cleaners	9111	359	1.53	1.34 to 1.74
Heavy truck and lorry drivers	8332	212	1.52	1.29 to 1.79
Security guards	5414	61	1.50	1.12 to 2.00
Manufacturing labourers NEC	9329	84	1.50	1.17 to 1.92
Building caretakers	5153	168	1.49	1.25 to 1.79
Fitness and recreation instructors	3423	61	1.48	1.11 to 1.97
Welders and flame cutters	7212	55	1.46	1.08 to 1.98
Food and related products machine operators	8160	55	1.45	1.07 to 1.96
Social work associate professionals	3412	97	1.43	1.14 to 1.80
Employment agents and contractors	3333	59	1.40	0.78 to 1.40
Motor vehicle mechanics and repairers	7231	112	1.40	1.12 to 1.73
Teaching professionals NEC	2359	73	1.38	1.06 to 1.79
Shop sales assistants	5223	644	1.37	1.23 to 1.52
Police officers	5412	61	1.36	1.02 to 1.81
Managing directors	1120	62	1.35	1.02 to 1.80
Early childhood educators	2342	321	1.32	1.15 to 1.51
Gardeners, horticulturalists and nursery growers	6113	53	1.29	0.95 to 1.75
Regulatory government professionals NEC	3359	51	1.28	0.94 to 1.73
Social work and counselling professionals	2635	123	1.27	1.04 to 1.56
Data entry clerks	4132	233	1.27	1.09 to 1.48
Professional services mangers	1349	71	1.26	0.97 to 1.64
Shopkeepers	5221	83	1.23	0.97 to 1.57
Assemblers NEC	8219	86	1.23	0.97 to 1.56
Secondary education teachers	2330	93	1.22	0.97 to 1.54
Contact centre information clerks	4222	89	1.22	0.96 to 1.54
Cooks	5120	100	1.21	0.97 to 1.51
Building frame and related trade workers NEC	7119	62	1.21	0.92 to 1.61
Accounting and bookkeeping clerks	4311	103	1.20	0.96 to 1.49
Waiters	5131	64	1.20	0.91 to 1.59
Health service managers	1342	55	1.19	0.88 to 1.59
Production clerks	4322	270	1.19	1.03 to 1.37
Primary school teachers	2341	328	1.17	1.02 to 1.33
Metal-working machine tool setters	7223	137	1.17	0.96 to 1.42
University and higher education teachers	2310	109	1.16	0.94 to 1.44
Policy administration officials	2422	213	1.14	0.97 to 1.33
Physical and engineering technicians NEC	3119	65	1.12	0.85 to 1.46
Agricultural and industrial machinery mechanics and repairers	7233	69	1.09	0.84 to 1.43
Engineering professionals	2149	60	1.07	0.81 to 1.42
Civil engineers	3112	50	1.07	0.78 to 1.45
House builders	7111	103	1.06	0.88 to 1.28
Credit and loans officers	3312	73	1.05	0.81 to 1.86
Business services agents NEC	3339	55	1.05	0.78 to 1.40
Personnel clerks	4416	62	1.00	0.76 to 1.32
Mechanical engineers	3115	86	0.99	0.78 to 1.25
Plumbers	7126	94	0.98	0.78 to 1.24
Electricians	7411	75	0.98	0.76 to 1.26
Electrical engineers	3113	51	0.96	0.71 to 1.30

OR are shown with 95% CI relative to occupations with low exposure in a national case–control study covering 1 July 2006 to 31 December 2019.*

*The reference group was defined as occupations classified with the lowest level of potential exposure to influenza using the JEMs.

ISCO, International Classification of Occupations; JEMs, job-exposure matrices; NEC, not elsewhere classified.

Elementary workers not elsewhere classified includes those who issue and collect parking or admission tickets, provide personal items to customers in cloakrooms and assist at entertainment events.

## Discussion

In this study with national coverage, we show that close contact with infected or diseased patients/persons and close physical proximity increase the odds of having a positive test for influenza virus. The observed pattern among the occupations supports the notion that contact with infected patients/persons and close proximity are important risk factors: primary care physicians, protective service workers, medical and laboratory technicians, taxi drivers, bus and tram drivers, home-based personal care workers, personal care workers, teachers’ aides and healthcare assistants were the occupations with the highest odds for influenza.

A major strength of our study design is that we use a national database with high coverage to assess the outcome of interest: influenza virus infection. The SmiNet registry provides comprehensive coverage of all influenza tests, although we acknowledge that not all cases with positive virus detection will be captured. We also use the Swedish Inpatient Register, which is acknowledged to be of high quality.^22^ Another strength of our study is that we employ random controls from the same national population. Furthermore, we were able to consider a number of key potential confounders using Swedish registry data. These confounders include level of education (as a proxy for socioeconomic status, SES) and comorbidities that may modify the influenza risk, such as diabetes, COPD, IHD and dispensed corticosteroids.

A key analytical strength of this study is our approach of categorising occupational exposure. It is generally acknowledged that the JEM approach avoids the recall bias inherent to respondent-elicited exposure histories. Furthermore, we limit the analyses to occupational exposures during the year preceding the diseased state, as we assume that this period is critical for increased risk. The Swedish JEM for proximity and exposure to diseases is based on prepandemic survey data collected in the USA. Noteworthy is that US military personnel were not included in these data. As military personnel make up a very small fraction of the Swedish working-age population, we consider that difference to be of minor importance. We do not include the dimension of face covering, which may have been used differently during the pandemic period and our study period. In addition, we do not include the dimensions of income insecurity or migrant background. The reason for this is twofold. First, we have individual data on socioeconomic status and migrant status on individual level from our national registries. Second, we use the Danish application of the European JEM, and we consider that the Swedish and Danish labour markets are different with regard to migration and income security. The reason for using two different JEMs was a matter of validity (external) of the results. We think, as both JEMs, points towards social distancing and physical proximity as important risk factors, strengthen the validity of our results.

Our study has a number of limitations. First, we do not control for influenza vaccination. However, in the group aged <65 years, we assume that a low fraction of the individuals has been vaccinated. The official Swedish recommendation is that individuals aged >65 years should be vaccinated, as well as individuals with COPD, IHD, diabetes and compromised immunity (www.folkhalsomyndigheten.se). In addition, HCWs are also recommended to be vaccinated, and especially in the healthcare sector, the staff may have been invited to receive free vaccinations for influenza. Another weakness is that we do not adjust for household size. This may be a confounder, as large households have been associated with increased risk, and occupations at risk, such as taxi drivers and HCWs, may live in larger households.^8^ We have adjusted for country of birth and SES, which to some extent take this bias into account. Another possible cofounder is the use of public transportation. A notable weakness of our study is the lack of data on smoking habits, in particular current tobacco use. Current smokers have a fivefold increased risk for laboratory-confirmed influenza, as compared with non-smokers.^23^ In Sweden, the prevalence of current smoking in the age group 50–65 years is approximately 17%.^24^ Hence, smoking may be a potential confounder that is sufficiently common to explain the associations that we observed, if compared with others, current smokers were substantially more likely than never-smokers to work the exposed occupations. In a German study, HCWs had a higher prevalence of current smoking compared with non-HCW.^8^ Of note, we have adjusted for both COPD and educational level, which may diminish the smoking bias. It is also important to point out that we are studying cases with a positive test for influenza.

The Dutch version of European JEM was validated against questionnaire data, and the agreement was good with weighted κ≥0.70 for number of contacts, nature of contacts, contaminated workspaces and social distance.^25^

It is also worth mentioning that we performed the JEM analyses based on assumptions of exposure to infected patients/persons and the risk of close proximity. The subsequent analyses of a high number of occupations may have given some spurious associations, although the pattern of risky occupations support the results of our exposure analysis. Instead, we may have missed some occupations at risk, due to small numbers, that is, occupations with fewer than 50 infected persons.

Our findings are broadly consistent with the report results from smaller studies conducted in other countries and contexts. Our results corroborate earlier studies of increased risk among HCWs, even if there are studies showing the opposite.^6–9^ It is also noteworthy that HCWs such as psychologists, dentists and midwives are not among those with increased risk, which may simply reflect the low numbers of these HCWs. However, we conclude that close contact with other persons and/or work with infected patients/persons considerably increase the risk for influenza. Other groups that have close contacts with both the general public and infected persons are taxi drivers and bus drivers. In a Chinese study, using a taxi more than once a week was a clear risk for severe acute respiratory syndrome (SARS-CoV-1).^26^ Infected persons may use public transportation; a British study noted that during the influenza season of 2008–2009, patients attending their primary care physician more often had used the bus or tram prior to their physician contact, as compared with controls.^27^ This may represent a way for bus/tram drivers to be infected. It is of interest that we found minor increases in odds among teachers, both academic teachers and primary school teachers, which is in line with the findings from the COVID-19 pandemic. The findings of our study are similar to the findings obtained in many recent studies investigating the occupational risks for COVID-19.^15 28^ HCWs and occupations that involve close contact to other persons, such as taxi drivers and hairdressers, are at increased risk. Of note, we did not observe any substantial difference between the odds for influenza A and influenza B in any of the analysed dimensions. It should be stressed that in the present study, we did not cover the pandemic COVID-19 period. That period merits a separate study to investigate the interplay between the influenza and SARS-CoV-2.

Our results indicate that occupational groups with frequent contact with the public, as well as HCW are at increased risk for influenza. The HCW should be further strongly encouraged to complete vaccinations. In addition, it should be considered whether other groups with frequents public contact such as teachers, taxi drivers and bus drivers, also should be offered vaccinations.

We conclude that close contact with infected or diseased patients/persons, and close physical proximity increased the odds of having a positive test for influenza virus. The results are similar for both influenza A and influenza B. The findings for different occupations support the idea that contacts with infected patients/persons and close proximity are important risk factors. There is a need to introduce additional safety measures, such as extended vaccination programmes, to reduce viral transmission in these environments.

## Data Availability

Data are available on reasonable request. The study outcomes are based on matching with national registers, and requesters of information for sharing need to have a Swedish ethical application permit. The lead author (KT) affirms that the manuscript is an honest, accurate and transparent account of the study being reported; that no important aspects of the study have been omitted; and that any discrepancies from the study as planned have been explained.

## References

[1] Pujol J , Godoy P , Soldevila N , et al . Social class based on occupation is associated with hospitalization for a (H1N1) pdm09 infection. Comparison between hospitalized and ambulatory cases. Epidemiol Infect 2016;144:732–40. 10.1017/S0950268815001892 26271901

[2] Yamazaki Y , Goto N , Iwanami N , et al . Outbreaks of influenza B infection and pneumococcal pneumonia at a mental health facility in Japan. J Infect Chemother 2017;23:837–40. 10.1016/j.jiac.2017.07.014 28838778

[3] Sansone M , Wiman Å , Karlberg ML , et al . Molecular characterization of a nosocomial outbreak of influenza B virus in an acute care hospital setting. J Hosp Infect 2019;101:30–7. 10.1016/j.jhin.2018.06.004 29909095 PMC7114871

[4] Tellier R . Review of aerosol transmission of influenza A virus. Emerg Infect Dis 2006;12:1657–62. 10.3201/eid1211.060426 17283614 PMC3372341

[5] Østergaard L , Mortensen RN , Kragholm K , et al . Work exposure and associated risk of hospitalisation with pneumonia and influenza: a nationwide study. Scand J Public Health 2021;49:57–63. 10.1177/1403494820964974 33124945 PMC7859585

[6] Lietz J , Westermann C , Nienhaus A , et al . The occupational risk of influenza A (H1N1) infection among healthcare personnel during the 2009 pandemic: a systematic review and meta-analysis of observational studies. PLOS One 2016;11:e0162061. 10.1371/journal.pone.0162061 27579923 PMC5006982

[7] Bandaranayake D , Huang QS , Bissielo A , et al . Risk factors and immunity in a nationally representative population following the 2009 influenza A (H1N1) pandemic. PLoS One 2010;5:e13211. 10.1371/journal.pone.0013211 20976224 PMC2954793

[8] Williams CJ , Schweiger B , Diner G , et al . Seasonal influenza risk in hospital healthcare workers is more strongly associated with household than occupational exposures: results from a prospective cohort study in Berlin, Germany, 2006/07. BMC Infect Dis 2010;10:8. 10.1186/1471-2334-10-8 20067628 PMC2836320

[9] Hudson B , Toop L , Mangin D , et al . Pandemic influenza A (H1N1) pdm09: risk of infection in primary healthcare workers. Br J Gen Pract 2013;63:e416–22. 10.3399/bjgp13X668212 23735413 PMC3662459

[10] Seale H , Weston KM , Dwyer DE , et al . The use of oseltamivir during an influenza B outbreak in a chronic care hospital. Influenza Other Respir Viruses 2009;3:15–20. 10.1111/j.1750-2659.2008.00063.x 19453437 PMC4941909

[11] Pujol J , Godoy P , Soldevila N , et al . Effect of occupational exposure on a (H1N1) pdm09 infection and hospitalization. Ann Occup Hyg 2016;60:1009–19. 10.1093/annhyg/mew044 27432191

[12] Carlsten C , Gulati M , Hines S , et al . COVID-19 as an occupational disease. Am J Ind Med 2021;64:227–37. 10.1002/ajim.23222 33491195 PMC8014565

[13] Henneberger PK , Cox-Ganser JM , Guthrie GM , et al . Estimates of COVID-19 vaccine uptake in major occupational groups and detailed occupational categories in the United States, April-May 2021. Am J Ind Med 2022;65:525–36. 10.1002/ajim.23370 35587657 PMC9348117

[14] Beale S , Patel P , Rodger A , et al . Occupation, work-related contact and SARS-cov-2 anti-nucleocapsid serological status: findings from the virus watch prospective cohort study. Occup Environ Med 2022;79:729–35. 10.1136/oemed-2021-107920 35450951 PMC9072780

[15] Bonde JPE , Sell L , Flachs EM , et al . Occupational risk of COVID-19 related hospital admission in Denmark 2020–2021: a follow-up study. Scand J Work Environ Health 2023;49:84–94. 10.5271/sjweh.4063 36228167 PMC10549918

[16] Oude Hengel KM , Burdorf A , Pronk A , et al . Exposure to a SARS-cov-2 infection at work: development of an international job exposure matrix (COVID-19-JEM). Scand J Work Environ Health 2022;48:61–70. 10.5271/sjweh.3998 34788471 PMC8729167

[17] Alderling M , Albin M , Ahlbom A , et al . Risk ATT sjukhusvårdas för covid-19 I olika yrken. Rapport 2021:02. [in Swedish]. Stockholm, Sweden Centrum för Arbets- och miljömedicin; 2021.

[18] Torén K , Albin M , Alderling MN , et al . Transmission factors and exposure to infections at work and invasive pneumococcal disease. Am J Ind Med 2023;66:65–74. 10.1002/ajim.23439 36385261 PMC10100104

[19] Torén K , Blanc PD , Naidoo RN , et al . Occupational exposure to dust and to fumes, work as a welder and invasive pneumococcal disease risk. Occup Environ Med 2020;77:57–63. 10.1136/oemed-2019-106175 31848233 PMC7029234

[20] ILO . International standard classification of occupations, ISCO-88. Geneva, 1990.

[21] ILO . International standard classification of occupations, ISCO-08. Geneva, 2012.

[22] Ludvigsson JF , Andersson E , Ekbom A , et al . External review and validation of the Swedish national inpatient register. BMC Public Health 2011;11:450. 10.1186/1471-2458-11-450 21658213 PMC3142234

[23] Lawrence H , Hunter A , Murray R , et al . Cigarette smoking and the occurrence of influenza-systematic review. J Infect 2019;79:401–6. 10.1016/j.jinf.2019.08.014 31465780

[24] Toren K , Olin A-C , Lindberg A , et al . Vital capacity and COPD: the Swedish Cardiopulmonary bioimage study (SCAPIS). COPD 2016;11:927. 10.2147/COPD.S104644 PMC4859418 27194908

[25] van der Feltz S , Peters S , Pronk A , et al . Validation of a COVID-19 job exposure matrix (COVID-19-jem) for occupational risk of a sars-cov-2 infection at work: using data of dutch workers. Ann Work Expo Health 2023;67:9–20. 10.1093/annweh/wxac032 35583140 PMC9129190

[26] Wu J , Xu F , Zhou W , et al . Risk factors for SARS among persons without known contact with SARS patients, Beijing, China. Emerg Infect Dis 2004;10:210–6. 10.3201/eid1002.030730 15030685 PMC3322931

[27] Troko J , Myles P , Gibson J , et al . Is public transport a risk factor for acute respiratory infection? BMC Infect Dis 2011;11:16. 10.1186/1471-2334-11-16 21235795 PMC3030548

[28] Würtz AM , Kinnerup MB , Pugdahl K , et al . Healthcare workers’ SARS-cov-2 infection rates during the second wave of the pandemic: follow-up study. Scand J Work Environ Health 2022;48:530–9. 10.5271/sjweh.4049 35780381 PMC10539104

