# Supplementary material for: Occupational risks for infection with influenza A and B: a national case–control study covering 1 July 2006–31 December 2019
Source: Occup Environ Med. 2023 May 16;80(7):377–83. doi: 10.1136/oemed-2022-108755 (PMC10314001; doi:10.1136/oemed-2022-108755)
Supplement: Supplementary data [file oemed-2022-108755supp001.pdf]

BMJ Publishing Group Limited (BMJ) disclaims all liability and responsibility arising from any reliance placed on this supplemental material which has been supplied by the author(s)

Online Supplement

Occupational risks for infection with influenza A and B – a national case-control study covering July 1, 2006 to December 31, 2019

Kjell Torén, Maria Albin, Tomas Bergström, Magnus Alderling, Linus Schiöler, Maria Åberg

Table S1. Conditional logistic multivariable regression models of influenza matched for age, and geographic region, and adjusted for education, country of birth, COPD, IHD, diabetes and use of steroids in relation to the different dimensions of transmission and mitigation factors, stratified for gender (Model 2) in a national case-control study covering July 1, 2006 to December 31, 2019.

**Dimensions of transmission and mitigation factors**	**Influenza<br>Odds ratios with 95% confidence intervals[a]**	
	Men	Women
Number of workers in close proximity to each other[a]		
<10 per day	1.05 (0.97-1.13)	1.02 (0.93-1.11)
10-30 per day	1.14 (1.04-1.25)	1.15 (1.06-1.24)
>30 per day	1.30 (1.18-1.43)	1.25 (1.16-1.34)
Nature of contacts[a]		
In workspaces with coworkers only	1.04 (0.96-1.12)	1.06 (0.97-1.15)
In workspaces with general public	1.14 (1.04-1.24)	1.09 (1.01-1.17)
Regular contacts with infected patients	1.64 (1.46-1.85)	1.34 (1.25-1.45)
Contaminated workspaces[a]		
Frequently sharing material/surfaces with coworkers (≥10 times/day)	1.04 (0.96-1.12)	1.10 (1.01-1.20)
Sometimes sharing material/surfaces with general public (<10 times/day)	1.11 (0.94-1.30)	1.03 (0.91-1.18)
Frequently sharing materials/surfaces with general public (≥10 times/day)	1.26 (1.16-1.38)	1.20 (1.12-1.28)
Location[a]		
Mostly working outside	1.25 (1.02-1.55)	1.06 (0.71-1.59)
Working partly inside	1.08 (0.97-1.21)	1.04 (0.85-1.28)
Working mostly inside	1.12 (1.04–1.20)	1.17 (1.09-1.24)
Social distancing[a]		
Always maintained	1.07 (1.00-1.14)	1.02 (0.91-1.14)
Not always	1.11 (1.03-1.18)	1.10 (9.98-1.24)
Never maintained	1.31 (1.23-1.40)	1.25 (1.12-1.40)
Physical proximity		
3rd vs. 2nd and 1st	1.07 (1.00-1.15)	1.08 (1.01-1.17)
4th vs. 2nd and 1st	1.39 (1.26-1.52)	1.35 (1.25-1.46)
Exposure to diseases or infections		
2nd vs. 1st	1.13 (1.04-1.23)	1.15 (1.07-1.23)
3rd vs. 1st	1.22 (1.07-1.39)	1.15 (1.08-1.23)
4th vs. 1st	1.67 (1.43-1.94)	1.45 (1.34-1.57)

COPD=chronic obstructive pulmonary disease; IHD=ischemic heart disease;
a. Compared with homeworkers or working alone.

Table S2. Conditional logistic multivariable regression models of influenza with pneumonia in relation to the different dimensions of transmission and mitigation factors in a national case-control study covering July 1, 2006 to December 31, 2019.

**Dimensions of transmission and mitigation factors**	**Influenza pneumonia (N=7,458) Odds ratios with 95% confidence intervals**	
	Basic models (Model 1)	Adjusted models (Model 2)
Number of workers in close proximity to each other[c]		
<10 per day	1.17 (1.09-1.27)	1.05 (0.97-1.14)
10-30 per day	1.43 (1.32-1.55)	1.20 (1.10-1.31)
>30 per day	1.38 (1.28-1.49)	1.21 (1.12-1.32)
Nature of contacts[c]		
In workspaces with coworkers only	1.18 (1.09-1.27)	1.06 (0.98-1.15)
In workspaces with general public	1.32 (1.23-1.43)	1.16 (1.07-1.25)
Regular contacts with infected patients	1.58 (1.45-1.72)	1.28 (1.17-1.41)
Contaminated workspaces[c]		
Frequently sharing material/surfaces with coworkers (≥10 times/day)	1.15 (1.07-1.24)	1.06 (0.98-1.15)
Sometimes sharing material/surfaces with general public (<10 times/day)	1.08 (0.93-1.24)	1.05 (0.90-1.22)
Frequently sharing materials/surfaces with general public (≥10 times/day)	1.48 (1.38-1.59)	1.22 (1.13-1.32)
Location[c]		
Mostly working outside	1.67 (1.32-2.12)	1.26 (0.97.1.64)
Working partly inside	1.23 (1.09-1.39)	1.08 (0.95-1.23)
Working mostly inside	1.31 (1.23 – 1.40)	1.15 (1.07-1.23)
Social distancing[c]		
Always maintained	1.15 (1.07-1.24)	1.07 (0.99-1.16)
Not always	1.31 (1.21-1.42)	1.15 (1.05-1.25)
Never maintained	1.52 (1.41-1.64)	1.23 (1.13-1.34)
Physical proximity		
3rd vs. 2nd and 1st	1.15 (1.07-1.23)	1.08 (1.01-1.16)
4th vs. 2nd and 1st	1.47 (1.36-1.59)	1.24 (1.14-1.35)
Exposure to diseases or infections		
2nd vs. 1st	1.34 (1.26-1.44)	1.16 (1.08-1.25)
3rd vs. 1st	1.32 (1.22-1.43)	1.21 (1.11-1.32)
4th vs. 1st	1.09 (0.98-1.21)	1.05 (0.94-1.18)

BMJ Publishing Group Limited (BMJ) disclaims all liability and responsibility arising from any reliance placed on this supplemental material which has been supplied by the author(s)

a. Matched for gender, age, geographic region;
b. Matched for gender, age, geographic region and adjusted for education, country of birth, COPD, IHD, diabetes and use of steroids; c. Compared with homeworkers or working alone.
